# Kaempferol promotes melanogenesis and reduces oxidative stress in PIG1 normal human skin melanocytes

**DOI:** 10.1111/jcmm.17711

**Published:** 2023-03-15

**Authors:** Yihui Xie, Xingyu Mei, Weimin Shi

**Affiliations:** ^1^ Department of Dermatology, The Affiliated Hospital of Jiaxing University The First Hospital of Jiaxing Jiaxing China; ^2^ Department of Dermatology, Shanghai General Hospital Shanghai Jiao Tong University School of Medicine Shanghai China

**Keywords:** apoptosis, kaempferol, melanin, PIG1 normal human skin melanocytes, ROS

## Abstract

Vitiligo is an autoimmune disease characterized by depigmentation. Kaempferol is a flavonoid compound with broad anti‐inflammatory and antioxidant properties. The purpose of this study was to investigate the effect of kaempferol on melanogenesis in PIG1 normal human skin melanocytes and its response to oxidative stress. The effect of kaempferol on melanin synthesis in PIG1 normal human skin melanocytes was explored by measuring tyrosinase activity, melanin content, mRNA and protein expression of key enzymes and expression of related pathway proteins. The effects of kaempferol pretreatment on cell viability, apoptosis, ROS level and HO‐1 protein level under H_2_O_2_ stimulation were explored. When treated with kaempferol, the tyrosinase activity and melanin content of PIG1 cells increased, the mRNA and protein expressions of TYR, TRP1, TRP2 and MITF increased, and the phosphorylation level of ERK1/2 increased. Upon the stimulation of H_2_O_2_, kaempferol reduced the production of ROS, decreased apoptosis and increased the protein expression of HO‐1 in PIG1 cells. In addition, kaempferol inhibited oxidative stress‐induced melanin reduction and promoted melanin synthesis in PIG1 cells and protected against H_2_O_2_‐induced oxidative stress damage.

## 1 INTRODUCTION

As a common depigmented skin disease, vitiligo has a prevalence of 0.5%–2% of the world population, which will have a great negative impact on the daily life of patients.^1^ Vitiligo is a multifactorial disease characterized by the loss of functional melanocytes, mainly including genetics, autoimmune responses, oxidative stress, production of inflammatory mediators and melanocyte detachment mechanisms.^2^ Oxidative stress is considered to be the most critical trigger of vitiligo. In response to stress, melanocytes release reactive oxygen species (ROS), and excess ROS eventually leads to endoplasmic reticulum‐related melanocyte apoptosis. Once cells are under oxidative stress, the key transcription factor Nrf2 regulates the expression of antioxidant genes by binding to ARE sequences and promotes the transcription of downstream antioxidant enzymes such as haeme oxygenase‐1 (HO‐1). Loss of antioxidant capacity caused by defects in the Nrf2‐ARE pathway is a pathogenic mechanism characteristic of vitiligo patients.^3^

Kaempferol (KF) is a flavonoid found naturally in tea as well as many common vegetables and fruits, and KF is best known for its antioxidant and anti‐inflammatory properties.^4^ KF has been shown to have beneficial effects on both chronic and acute inflammatory diseases.^5^, ^6^ The second important use of KF is cancer prevention.^7^ KF is non‐toxic and inexpensive and has great economic value.^8^ Regulated cell death ensures that the homeostasis of the organism is maintained. The endoplasmic reticulum is a key organelle involved in protein homeostasis, and kaempferol as a flavonoid may regulate endoplasmic reticulum stress and autophagy and have protective effects on dysfunctional cells.^9^

By analysing the relationship between the chemical structure of flavonoids and promoting melanogenesis, existing studies infer that phenyl group play an important role in stimulating melanogenesis, so we infer that KF has a role in promoting melanin synthesis.^10^

Photochemotherapy using 8‐methoxypsoralen (8‐Mop) and long‐wave ultraviolet radiation is commonly used to treat vitiligo.^11^ The main potential side effects of topical photochemotherapy are uncontrolled light reactions manifested by severe sunburn, blisters and abnormally dark pigmentation.^12^ Animal experiments and long‐term follow‐up studies of photochemotherapy patients have shown that photochemotherapy has a certain carcinogenic potential, and the mutagenicity of photochemotherapy in microorganisms and cell cultures has also been studied.^13^

The exploration of vitiligo treatment has never stopped. Our research explored the effect of KF on PIG1 normal human skin melanocytes, providing more research basis and new directions for drug development for KF in the treatment of depigmented skin diseases such as vitiligo.

## 2 MATERIAL AND METHODS

### 2.1 Cell culture and treatment

PIG1 normal human skin melanocytes (Otwo Biotech, Shenzhen, China) were cultured in high glucose DMEM (Gibco) supplemented with 10% fetal bovine serum (Gibco) at 37°C in 5% carbon dioxide. KF (Aladin) was dissolved in DMSO, and the final concentration of DMSO in the medium was one‐thousandth.

### 2.2 Cell viability

Cell viability was determined using the Cell Counting Kit‐8 (NovaBio). PIG1 was inoculated in a 96‐well plate at a density of 5000/well, treated with KF after adhering, and CCK‐8 solution added according to the instructions. After incubation for 1 h, the microplate reader measured the absorbance at 450 nm.

### 2.3 Tyrosinase activity measurements

After KF treatment, the culture medium was discarded, the cells washed three times with PBS, cell lysate added, lysed on ice for 30 min and then it was scraped off with a cell scraper and then put into an EP tube at 12000 rpm for 5 min at 4°C; then, the supernatant was taken and 500 μl L‐DOPA (1 mM) added; 200 μl/well was added to the 96‐well plate, and after incubation at 37°C for 3 h, the absorbance was measured at 475 nm with a microplate reader.

### 2.4 NaOH assay of melanin content

After KF treatment, the cells were collected, and 1 mL of 1 M NaOH was added and then the solution was heated at 80°C for 1 h, and 200 μl/well was added to a 96‐well plate. The microplate reader measured absorbance at 405 nm.

### 2.5 Quantitative polymerase chain reaction (qPCR)

After the cells were treated with KF, trizol was added to lyse the cells, shaken at room temperature for 15 s, kept undisturbed for 3 min, and centrifuged at 12000 rpm, 4°C for 15 min. The upper colourless water sample layer was taken, an equal volume of isopropanol was added and mixed well; the mixture was kept undisturbed at room temperature for 10 min, centrifuge at 12,000 rpm for 10 min at 4°C and 1 mL of 75% ethanol was added to wash and then centrifuged at 17,108 *g* for 5 min at 4°C and the supernatant discarded, and finallyair dried for 3 min, DEPC100UL added and stored at −80°C. RNA was reverse transcribed into cDNA using a reverse transcription kit (NovaBio R202) according to the manufacturer's instructions. Target gene expression was assayed using SYBR qPCR mix (NoviaBio Q204) according to the manufacturer's instructions.

### 2.6 Protein extraction and western blotting

Cells were cultured and processed in 10‐mm dishes. The cells were discarded from the culture medium, washed three times with PBS, added with 500 μl of cell lysis solution (RIPA: PMSF = 100:1), lysed on ice for 30 minutes, scraped off with a cell scraper and placed in an EP tube, centrifuged at 4°C and 12,000 rpm for 5 min, and the supernatant was taken to a new EP tube, added with Ladding buffer (supernatant: buffer = 3:1), heated at 100°C for 10 min and stored in a −80°C refrigerator.

Sodium dodecyl sulphate‐polyacrylamide gel electrophoresis (SDS‐PAGE) was performed on each extracted sample, and proteins were transferred to polyvinylidene fluoride membranes using a semidry blotter (Bio‐Rad Laboratories). Membranes were blocked in rapid blocking solution (New cell and molecular biotech) for 1 h and incubated with primary antibody overnight at 4°C. After washing three times with TBST, the cells were incubated with HRP‐conjugated secondary antibody for 1 h and then developed using a high‐sensitivity ECL chemiluminescence detection kit (E412‐01, Vazyme).

Antibody:TYR (R 1:1000 ab170905, abcam), TRP1 (R 1:10000 ab178676, abcam), TRP2 (R 1:500 Cat No.13095‐1‐AP, proteintech), MITF (R 1:200 ab140606, abcam), ERK (R 1:1000 16,443‐1‐AP, proteintech), PERK (R 1:100028733‐1‐AP, proteintech), AKT (R 1:2000,60,203‐2‐lg, proteintech), PAKT (R 1:2000 66,444‐1‐lg), HO‐1(R 1:10000 ab68477, abcam), GAPDH (M 1:20000 Cat No. 60004‐1‐Ig, proteintech), β‐ACTIN (M 1:20000, Cat no: 66009‐1‐Ig, proteintech) and α‐TUBULIN (M 1:20000, Cat no: 11224‐1‐AP, proteintech).

### 2.7 Immunofluorescence (IF)

Cells were plated in six‐well plates, and after treatment, they were washed three times with PBS, fixed with 4% formaldehyde for 20 min and then washed three times with PBS. Cells were stained with antibodies and washed three times with PBS. Nuclei were stained with DAPI fluorescent dye and then washed three times with PBS and observed under an inverted fluorescence microscope.

### 2.8 Cell apoptosis measurement

After the cells were treated with KF for 24 h, they were stimulated with H_2_O_2_ for 1 h. Cells were trypsinized without EDTA and collected in tubes, then cells were washed twice with PBS and resuspend in 100 μL of PBS. According to the kit instructions, cells were stained with Annexin V‐FITC/PI apoptosis kit and detected by flow cytometry.

### 2.9 Intracellular ROS measurement

After 24 h of KF treatment, cells were stimulated with H_2_O_2_ for 1 h. We washed the cells twice with PBS to remove all medium and FBS and then diluted the DCFH‐DA probe to 1:1000 with medium and added it to each well. We incubated cells at 37°C for 30 min and then washed them three times with a serum‐free medium. Fluorescence was observed and recorded under an inverted fluorescence microscope and then the fluorescence intensity was measured using ImageJ. Cells were trypsinized without EDTA and collected in tubes for detection by flow cytometry, and FlowJo software was used to analyse cell mean fluorescence intensity.

### 2.10 Statistical analysis

Data in this work are presented as mean ± standard deviation (SD), and statistical analysis was performed using GraphPad Prism (version 6.0) Student's *t*‐test or one‐way analysis of variance (anova) for multiple group comparisons. Values of *p* < 0.05 were considered significant. All experiments were repeated at least three times.

## 3 RESULTS

### 3.1 KF promoted melanogenesis in PIG1 cells

**FIGURE 1:**
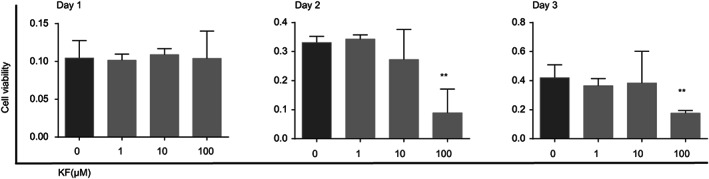
Cytotoxicity of KF on PIG1 cells. Different concentrations (0, 1, 10, 100 μM) of KF were treated with KF for 1, 2 and 3 days, and cell viability was determined by CCK8 assay.

At the outset of the experiment, we investigated the non‐toxic extent of KF to PIG1 cells using the CCK8 assay. PIG1 cells were treated with different concentrations (0, 1, 10, 100 μM) of KF for 1, 2 and 3 days. KF concentrations <100 μM did not affect PIG1 cells viability; 100 μM concentration significantly inhibited cell viability on the second and third days of treatment (Figure 1).

**FIGURE 2:**
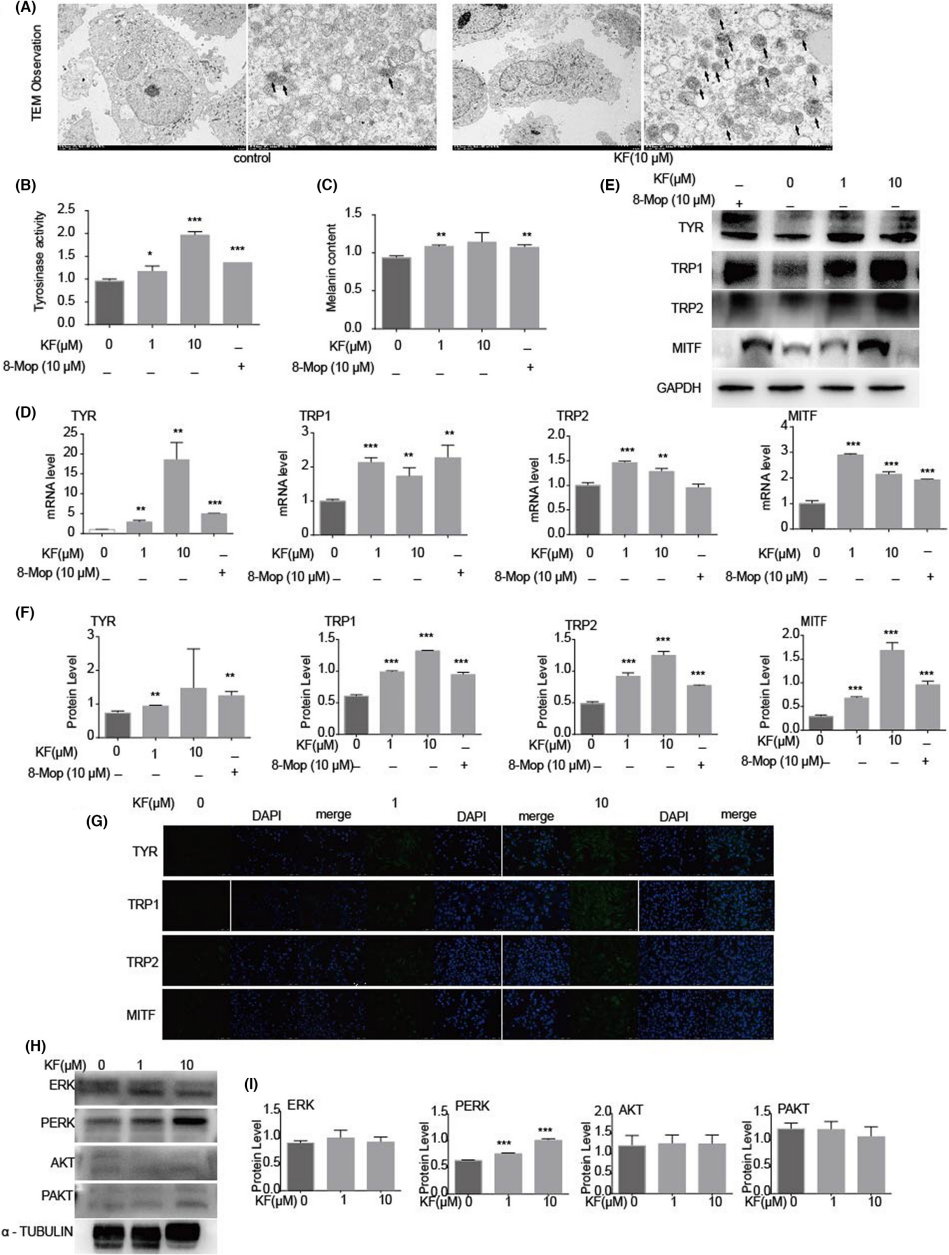
KF promoted melanin synthesis. (A) Under TEM, melanosomes in control group and KF group (10 μM, 24 h). Arrows mark stages III and IV mature melanosomes. (B) Tyrosinase activity of PIG1 cells after treatment with different concentrations (0, 1, 10 μM) of KF and 8‐Mop (10 μM) for 24 h. (C) Melanin content of PIG1 cells after treatment with different concentrations (0, 1, 10 μM) of KF and 8‐Mop (10 μM) for 24 h. (D) The mRNA expression levels of TYR, TRP1, TRP2 and MITF in PIG1 cells after treatment with different concentrations (0, 1, 10 μM) of KF and 8‐Mop (10 μM) for 24 h. (E) The protein expression levels of TYR, TRP1, TRP2 and MITF in PIG1 cells after treatment with different concentrations (0, 1, 10 μM) of KF and 8‐Mop (10 μM) for 24 h. (F) The grey value statistics of protein expression levels of TYR, TRP1, TRP2 and MITF in PIG1 cells after different concentrations (0, 1, 10 μM) of KF and 8‐Mop (10 μM) were treated for 24 h. (G) Fluorescence of TYR, TRP1, TRP2 and MITF in PIG1 cells after treatment with different concentrations (0, 1, 10 μM) of KF for 24 h. (H) The protein expression levels of ERK1/2, PERK1/2, AKT and PAKT in PIG1 cells after treatment with different concentrations (0, 1, 10 μM) of KF for 24 h. (I) The grey value statistics of the protein expression levels of ERK1/2, PERK1/2, AKT and PAKT in PIG1 cells after treatment with different concentrations (0, 1, 10 μM) of KF for 24 h.

We explored the effect of KF on melanogenesis. Under transmission electron microscopy (TEM), the number of dark stages III and IV melanosomes was significantly increased in PIG1 cells treated with KF (10 μM) for 24 h compared with the control group; light‐coloured immature stages I and II melanosomes are rare (Figure 2A).

On this basis, we compared tyrosinase activity (Figure 2B) and melanin content (Figure 2C). After KF treatment for 24 h, it was found that the tyrosinase activity and melanin content of cells increased in a concentration‐dependent manner, and the increase was not lower than the same concentration of 8‐Mop.

It is recognized that TYR, TRP1, TRP2 and MITF play a crucial role in melanosome pigmentation, so we explored their mRNA and protein expression. qPCR experiments showed that KF upregulated the mRNA expressions of TYR, TRP1, TRP2 and MITF, and the upregulation of TYR, TRP2 and MITF was higher than that of 8‐Mop at the same concentration (Figure 2D). Meanwhile, at the protein level, the expressions of TYR, TRP1, TRP2 and MITF were significantly increased in a concentration‐dependent manner (Figure 2 E,F). IF showed the same trend as protein expression (Figure 2G).

### 3.2 KF activated the MAPK (ERK1/2) signalling pathway in PIG1 cells

To explore the mechanism by which KF promotes melanogenesis, we compared the phosphorylation and total levels of ERK1/2 in MAPK signalling pathways and AKT after treating PIG1 cells with different concentrations (0, 1, 10 μM) of KF for 24 h. The results showed that the total level of ERK1/2 was consistent after KF treatment, and the level of p‐ERK increased in a concentration‐dependent manner; the protein levels of AKT and PAKT did not change (Figure 2H,I). From this, we concluded that KF activated the MAPK (ERK1/2) pathway in PIG1 cells, but did not affect the AKT pathway.

### 3.3 KF reduced H_2_O_2_ ‐induced apoptosis

**FIGURE 3:**
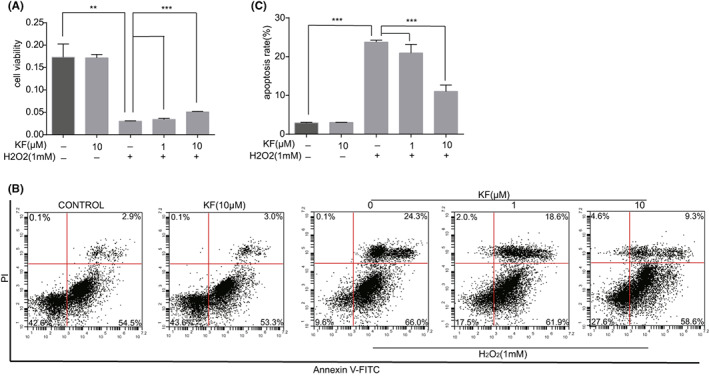
KF reduced apoptosis induced by H_2_O_2_. (A) Different concentrations (0, 1, 10 μM) of KF were pretreated for 24 h and then stimulated with H_2_O_2_ (1 mM) for 1 h, and the cell viability of PIG1 cells was determined by CCK8 method. (B) Different concentrations (0, 1, 10 μM) of KF were pretreated for 24 h and then stimulated with H_2_O_2_ (1 mM) for 1 h, and the apoptosis of PIG1 cells was measured by flow cytometry. (C) Different concentrations (0, 1, 10 μM) of KF were pretreated for 24 h and then stimulated with H_2_O_2_ (1 mM) for 1 h. The statistics of the apoptosis rate of PIG1 cells by flow cytometry.

We used H_2_O_2_ to simulate the oxidative stress state of cells and explored the role of KF in cellular oxidative stress. We pretreated PIG1 cells with KF (0, 1, 10 μM) for 24 h, followed by stimulation with 1 mM H_2_O_2_ for 1 h. Next, we measured the cell viability of the cells in each group. The cell viability after H_2_O_2_ treatment was significantly decreased. At the same time, the cell viability in the KF pretreatment group was significantly higher than that in the pure H_2_O_2_ treatment group. Therefore, pretreatment with KF enhanced cell viability under oxidative stress (Figure 3A). The measurement of cell apoptosis by flow cytometry showed that cell apoptosis was significantly increased after hydrogen peroxide treatment, the apoptosis rate in the KF pretreatment group was significantly lower than that in the pure H_2_O_2_ group, and KF had a protective effect on apoptosis caused by oxidative stress (Figure 3B,C).

### 3.4 KF reduced H_2_O_2_ ‐induced intracellular ROS production by upregulating HO‐1

**FIGURE 4:**
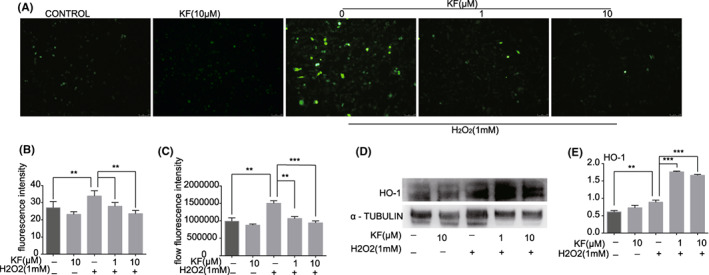
KF reduced ROS generation in PIG1 cells stimulated by H_2_O_2_. (A) Different concentrations (0, 1, 10 μM) of KF were pretreated for 24 h and then stimulated with H_2_O_2_ (1 mM) for 1 h. The ROS fluorescence of PIG1 cells was observed under an inverted fluorescence microscope. (B) Different concentrations (0, 1, 10 μM) of KF were pretreated for 24 h and then stimulated with H_2_O_2_ (1 mM) for 1 h. Statistical chart of ROS fluorescence of PIG1 cells under an inverted fluorescence microscope. (C) Different concentrations (0, 1, 10 μM) of KF were pretreated for 24 h and then stimulated with H_2_O_2_ (1 mM) for 1 h. The mean fluorescence intensity of ROS in PIG1 cells was analysed by flow cytometry. (D) Different concentrations (0, 1, 10 μM) of KF were pretreated for 24 h and then stimulated with H_2_O_2_ (1 mM) for 1 h, the protein expression of HO‐1 in PIG1 cells. (E) Different concentrations (0, 1, 10 μM) of KF were pretreated for 24 h, and then stimulated with H_2_O_2_ (1 mM) for 1 h, and the grey value statistics of PIG1 protein expression.

Reactive oxygen species generation is highly correlated with hydrogen peroxide‐induced apoptosis. We labelled intracellular ROS in PIG1 cells with a DCFH‐DA fluorescent probe and visualized and quantified ROS using an inverted fluorescence microscope and flow cytometry. Under the inverted fluorescence microscope, the cells in the control group showed no obvious fluorescence, and the cells stimulated with hydrogen peroxide showed a lot of fluorescence, while the fluorescence in the KF pretreatment group was significantly lower than that in the pure H_2_O_2_ group. At the same time, we used ImageJ to quantify the fluorescence intensity of the images (Figure 4A,B). Flow cytometry was used to detect ROS, and FlowJo software statistics of the mean fluorescence intensity of ROS also showed the same result (Figure 4C).

To explore the mechanism of KF scavenging ROS, we detected the level of HO‐1 protein in PIG1 cells. We found that there was no significant difference between the KF group and the control group. Under the stimulation of H_2_O_2_, the expression of the HO‐1 protein was significantly increased, and the expression of HO‐1 protein in the cells of the KF pretreatment group was significantly higher than that of the pure H_2_O_2_ stimulation group (Figure 4D,E).

### 3.5 KF protects melanin reduction caused by H_2_O_2_

**FIGURE 5:**
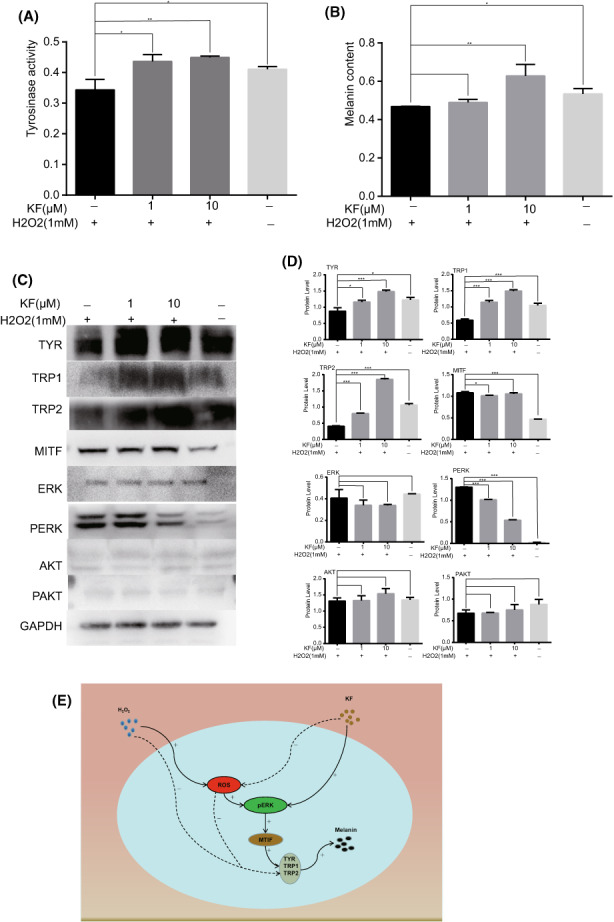
KF protected the reduction of melanin synthesis caused by H_2_O_2_. (A) Different concentrations (0, 1, 10 μM) of KF were pretreated for 24 h, and then stimulated with H_2_O_2_ (1 mM) for 1 h, the tyrosinase activity of PIG1 cells in different treatment groups. (B) Different concentrations (0, 1, 10 μM) of KF were pretreated for 24 h, and then stimulated with H_2_O_2_ (1 mM) for 1 h, the melanin content of PIG1 cells of PIG1 cells in different treatment groups. (C) Different concentrations (0, 1, 10 μM) of KF were pretreated for 24 h, and then stimulated with H_2_O_2_ (1 mM) for 1 h, the protein expression of TYR, TRP1, TRP2, MITF, ERK1/2, PERK1/2, AKT and PAKT in PIG1 cells. (D) Different concentrations (0, 1, 10 μM) of KF were pretreated for 24 h, and then stimulated with H_2_O_2_ (1 mM) for 1 h, the grey value statistics of TYR, TRP1, TRP2, MITF, ERK1/2, PERK1/2, AKT and PAKT in PIG1 cells. (E) KF inhibits oxidative stress while promoting melanin synthesis.

Existing studies have shown that excess H_2_O_2_ and ROS impair biological processes, including the key melanin synthase, thereby reducing melanin synthesis.^14^ At the same time, excess ROS can activate ERK, which has been verified in other articles^15^ Therefore, we wanted to explore the relationship between the protective effect of KF against oxidative stress and the promotion of melanin synthesis. We applied H_2_O_2_ stimulation to KF‐pretreated cells. H_2_O_2_ reduced the tyrosinase activity and melanin content of PIG1 cells, and the tyrosinase activity and melanin content of PIG1 cells pretreated with KF increased compared to those treated with H_2_O_2_ alone (Figure 5A,B). At the same time, the expression of TYR, TRP1 and TRP2 protein in the H_2_O_2_ treatment group decreased, and the protein expression of TYR, TRP1 and TRP2 in the KF pretreatment group increased compared with the pure H_2_O_2_ stimulation group. The phosphorylation level of ERK1/2 in the H_2_O_2_ treatment group was significantly increased, the phosphorylation level of ERK1/2 in the KF pretreatment group was decreased compared with that in the pure H_2_O_2_ treatment group, and the expressions of AKT and PAKT did not change significantly. However, the protein expression of MITF was consistent with the phosphorylation level of ERK1/2 (Figure 5C,D).

## 4 DISCUSSION

Our study found that KF promoted melanin synthesis in melanocytes by enhancing ERK1/2 phosphorylation and reduced apoptosis by inhibiting H_2_O_2_‐induced ROS generation by upregulating HO‐1 expression. At the same time, KF can protect against the melanin reduction caused by H_2_O_2_. Under the stimulation of H_2_O_2_, the effect of KF on melanin synthesis occurs through various pathways. As shown in the figure (Figure 5 E), H_2_O_2_ promotes the production of ROS to promote the phosphorylation of ERK1/2, thereby promoting the expression of MITF, TYR, TRP1 and TRP2, but at the same time, H_2_O_2_ can directly destroy the three enzymes TYR, TRP1 and TRP2, which will eventually lead to the reduction of melanin synthesis. KF promotes the phosphorylation of ERK1/2 and increases the synthesis of MITF, TYR, TRP1 and TRP2; at the same time, KF reduces the production of ROS, thereby reducing the phosphorylation of ERK1/2 and reducing the destruction of TYR, TRP1 and TRP2 by ROS eventually leading to an increase in melanin synthesis.

Existing studies have shown that kaempferol increases melanin content and dendritic length in melanoma cells and promotes cell migration.^16^ The effect of KF on normal human skin melanocytes has never been reported, and our study fills the gap of KF's role in promoting melanogenesis and inhibiting oxidative stress in normal human skin melanocytes.

Whether it is topical drug application, phototherapy or systemic treatment, vitiligo treatment is mainly to promote melanin production and reduce the loss of melanocytes^17^, ^18^; KF works in both ways and can be used as a new option for clinical drug development. As a natural flavonol, kaempferol has the characteristics of poor water solubility but strong lipophilicity, which is beneficial to the percutaneous absorption of drugs for local treatment and improves the utilization rate of drugs.^19^ At the same time, KF is a good metal ion chelating ligand, and the effect of KF can be enhanced by metal chelation.^20^

MAPK/ERK (mitogen‐activated protein kinases/extracellular signal‐regulated kinases) signalling is essential for the proliferation and differentiation of melanocytes.^21^ The kinase ERK in the MAPK signalling pathway is involved in the activation of the melanocyte receptor through ligand binding to its extracellular domain.^22^ By binding to its receptor, ligand activation leads to the upregulation of MITF.^23^ The PI3K/Akt (phosphatidylinositol 3′‐kinase/Akt) signalling pathway regulates the cell cycle through GSK‐3 and the protein cyclin D1 and plays a key role in melanocyte proliferation and apoptosis.^24^

ROS include oxygen radicals such as superoxide anion radicals and hydroxyl radicals and nonradical oxidants such as hydrogen peroxide and singlet oxygen.^25^ Under physiological conditions, the balance between ROS generation and ROS scavenging is highly controlled, and unregulated oxidative and reductive stress can lead to severe cellular damage and even unnecessary cell death, leading to whole organ and organismal failure.^26^ Oxidative stress is considered one of the possible pathogenic events responsible for melanocyte loss. The poor circulation of tetrahydrobiopterin in the epidermis of vitiligo patients may be highly correlated with the production of intracellular H_2_O_2_.^27^ Increased levels of ROS in melanocytes may lead to defective apoptosis leading to the release of abnormal proteins that can act as self‐antigens leading to autoimmunity.^28^

HO‐1 is considered a major protein in diseases caused by oxidative and inflammatory damage. It has become an important target protein that can be upregulated against various stressful events by regulating intracellular levels of pro‐oxidative haeme and other benefits conferred with by‐products such as carbon monoxide and biliverdin.^29^, ^30^ HO‐1 acts as a cytoprotective molecule against various forms of cell death, including apoptosis, necrosis and regulated cell death programmes (necroptosis, pyroptosis and ferroptosis).^31^ As an effective cytoprotective agent, the regulation of its expression level has potential therapeutic value in the treatment of vitiligo.^32^, ^33^ Hypoxia, oxidative stress, cytokines, LPS, heavy metals and a series of stimuli induce the production of HO‐1. Most HO‐1 inducers are widely found in flavourings, foods, spices and traditional medicinal plants.

MAPK is one of the most important signalling molecules associated with drug‐induced HO‐1.^34^, ^35^ In our study, KF not only upregulates the expression of melanin synthase through phosphorylation of ERK1/2 but also upregulates HO‐1 to reduce the production of ROS, thereby reducing the destruction of melanogenic enzymes by ROS.

Our experiments on KF in promoting melanin synthesis may have some defects. Yao Lu et al mentioned that KF can serve as a tyrosinase substrate,^36^ and meanwhile, Beata Gasowska‐Bajger et al showed that KF interferes with tyrosine determination.^37^ These problems make the results of tyrosinase activity and melanin content measured by absorbance inaccurate. However, the determination of mRNA and protein of melanogenic enzymes such as tyrosinase in our study can confirm the function of KF in promoting melanin synthesis from the lateral side. Inaccuracy for the absorbance measurement of tyrosinase activity and melanin synthesis led us to find experimental methods that are more specific for tyrosinase activity and melanin content assays.

Our research is the beginning of KF as a topical drug for vitiligo treatment, but the application of KF in vitiligo treatment requires further experiments and more optimized drug preparation, which requires the participation of multidisciplinary professionals such as pharmacology and chemistry.

## AUTHOR CONTRIBUTIONS

**Yihui Xie:** Conceptualization (equal); data curation (equal); formal analysis (equal); funding acquisition (equal); investigation (equal); methodology (equal); project administration (equal); resources (equal); software (equal); supervision (equal); validation (equal); visualization (equal); writing – original draft (equal); writing – review and editing (equal). **Xingyu Mei:** Conceptualization (equal); data curation (equal); formal analysis (equal); funding acquisition (equal); investigation (equal); methodology (equal); project administration (equal); resources (equal); software (equal); supervision (equal); validation (equal); visualization (equal); writing – original draft (equal); writing – review and editing (equal). **Weimin Shi:** Conceptualization (equal); data curation (equal); formal analysis (equal); funding acquisition (equal); investigation (equal); methodology (equal); project administration (equal); resources (equal); software (equal); supervision (equal); validation (equal); visualization (equal); writing – original draft (equal); writing – review and editing (equal).

## CONFLICT OF INTEREST STATEMENT

The authors confirm that there are no conflicts of interest.

## Data Availability

The data that support the findings of this study are available from the corresponding author upon reasonable request.
